# Biological Control of *Listeria monocytogenes* Growth in Fermented Buttermilk Enriched with Microfiltration Retentate

**DOI:** 10.3390/foods15101742

**Published:** 2026-05-14

**Authors:** Piotr Śmigiel, Jarosław Kowalik, Marika Bielecka

**Affiliations:** Department of Dairy Science and Quality Management, Faculty of Food Science, University of Warmia and Mazury, Oczapowskiego 7, 10-719 Olsztyn, Poland; j.kowalik@uwm.edu.pl (J.K.); marika.bielecka@uwm.edu.pl (M.B.)

**Keywords:** probiotics, acidified buttermilk, retentate, *Listeria monocytogenes*, *Lactobacillus acidophilus* LA-5^®^

## Abstract

The use of protective cultures and probiotic lactic acid bacteria is considered a potential strategy for controlling *Listeria monocytogenes* in food systems, particularly in minimally processed and fermented products. However, the behavior of foodborne pathogens in complex dairy matrices, especially those enriched with byproducts of milk processing, remains insufficiently characterized. The aim of this study was to evaluate the survival of *Listeria monocytogenes* in buttermilk enriched with retentate obtained after microfiltration, in the presence of the probiotic strain *Lactobacillus acidophilus* LA-5^®^. The study was conducted under different storage temperatures to reflect realistic conditions of product distribution and storage. The results demonstrated that fermented buttermilk with added retentate did not support the growth of *Listeria monocytogenes* under the tested conditions, and a gradual reduction in pathogen counts was observed during storage. The presence of *Lactobacillus acidophilus* LA-5^®^ was associated with a faster decrease in pathogen levels compared to samples without the probiotic strain. At the same time, lactic acid bacteria maintained high viability throughout the storage period. In contrast, predictive modelling using ComBase indicated the potential for pathogen growth under similar physicochemical conditions. This discrepancy highlights the limitations of predictive models when applied to complex, biologically active food matrices. These findings indicate that fermented buttermilk enriched with retentate may provide conditions limiting the survival of *L. monocytogenes*. However, the mechanisms responsible for the observed inhibition were not directly investigated in this study and require further research. The results emphasize the importance of experimental validation of predictive microbiology models and contribute to a better understanding of pathogen behavior in fermented dairy systems.

## 1. Introduction

*Listeria monocytogenes* is a highly pathogenic, Gram-positive, facultative anaerobic bacterium capable of growing over a wide temperature range, including under refrigerated conditions, which makes it a significant threat to food safety. Infection with this bacterium can lead to listeriosis—a disease with a varied clinical course, particularly dangerous for pregnant women, newborns, the elderly, and immunocompromised patients [1]. Although contamination of dairy products is not a common occurrence, its potential presence in ready-to-eat products poses a significant challenge for the dairy industry [2].

Buttermilk is a byproduct of butter production that is increasingly subjected to further processing, including fermentation with lactic acid bacteria. This product is characterized by a relatively low pH, high water activity, and the presence of easily digestible nutrients such as lactose, whey proteins, and minerals. These properties, on the one hand, determine its nutritional value and technological potential; on the other hand, they may support the survival of microorganisms under certain conditions [3,4].

In the event of secondary contamination, acidified buttermilk may provide conditions that allow the survival of *Listeria monocytogenes*, a pathogen capable of persisting under a wide range of environmental conditions, including refrigeration temperatures. While low temperature storage limits the growth of many microorganisms, it does not necessarily eliminate the risk associated with this pathogen. Therefore, the presence of *L. monocytogenes* in buttermilk remains a relevant concern for product safety [5,6].

For this reason, microbiological control of buttermilk during production, processing, and distribution is crucial for ensuring consumer safety. In recent years, increasing attention has been paid to alternative methods for limiting pathogen survival in dairy products, including biopreservation strategies based on the use of beneficial microorganisms. Buttermilk, due to its content of milk phospholipids and milk fat globule membrane (MFGM) components, may provide a favorable environment for the growth and metabolic activity of lactic acid bacteria. Selected lactic acid bacteria cultures may contribute to limiting the survival of *Listeria monocytogenes* through mechanisms such as acidification, competition for nutrients, and the production of antimicrobial metabolites, although these effects depend on the specific food matrix and conditions [7]. Lactic acid bacteria also play a role in maintaining a balanced microbial ecosystem by acting antagonistically toward pathogenic microorganisms [8].

The potential role of buttermilk-derived bioactive components in influencing *L. monocytogenes* behavior has also been suggested in previous studies. Sprong et al. [9] demonstrated that sweet buttermilk rich in MFGM components reduced colonization and translocation of *L. monocytogenes* in rats, most likely by inhibiting pathogen adhesion to intestinal mucosa rather than through direct bactericidal effects. These findings indicate that buttermilk components may affect pathogen behavior through mechanisms not fully explained by conventional physicochemical parameters alone.

In addition, some strains used in biocontrol exhibit documented probiotic properties, offering the possibility of improving both the microbiological safety and functional value of fermented dairy products. This approach is consistent with current trends in the sustainable utilization of dairy industry by-products and the development of foods with enhanced quality and safety.

Lactic acid bacteria strains, including *Lactobacillus acidophilus* LA-5^®^, are often classified as protective cultures and are known to exhibit antagonistic activity against various microorganisms, primarily through acidification and metabolite production [9,10]. However, the effectiveness of such cultures in complex dairy matrices, especially those enriched with processing by-products, requires further investigation.

In the context of buttermilk, it is particularly important to determine whether the presence of selected probiotic strains can influence the survival of *Listeria monocytogenes*, especially in technologically modified products such as protein-enriched buttermilk. At the same time, enrichment with microfiltration retentate increases nutrient availability and introduces bioactive components, which may potentially support microbial survival, making the net effect difficult to predict.

Challenges related to public health, economic losses associated with unsafe food, and increasing globalization have led the food industry to increasingly rely on tools that support food safety assessment. Predictive models used in food microbiology play an important role in this context.

Predictive models are widely used to describe and estimate the growth, survival, or inactivation of microorganisms under defined environmental conditions. They are based on mathematical relationships that allow prediction of changes in microbial populations over time and are commonly applied in the assessment of food safety during storage and distribution [11]. However, these models are typically developed using simplified systems and may not fully capture the complexity of real food matrices.

The hypothesis of this study was that the addition of microfiltration retentate modifies the microbial environment of fermented buttermilk and may influence the survival of *Listeria monocytogenes*, while the presence of a probiotic strain enhances inhibitory effects through microbial interactions.

The aim of this study was to evaluate the effect of *Lactobacillus acidophilus* LA-5^®^ on the survival of *Listeria monocytogenes* in buttermilk enriched with microfiltration retentate and to assess whether the use of protective cultures may contribute to limiting pathogen survival under the tested conditions. The study contributes to a better understanding of pathogen behavior in complex fermented dairy systems and provides data relevant to the application of predictive microbiology in such products.

## 2. Materials and Methods

### 2.1. Research Material

The experimental material was sweet pasteurized buttermilk obtained during butter production (SM Mlekovita Polska, Wysokie, Mazowieckie, Poland, Dairy Plant). Its basic chemical composition was as follows: fat content—0.84%, protein—3.07%, dry matter—9.52%, non-fat dry matter—8.51%, lactose—5.02%, which was analyzed using a MilkoScan™ FT3 device (Foss, Hillerød, Denmark).

To increase the protein content, the buttermilk was enriched with the retentate obtained after its membrane microfiltration. Microfiltration of the buttermilk was performed using ceramic membranes with a pore diameter of 0.1 µm and an effective filtration area of 0.72 m^2^ (three modules of 0.24 m^2^ each), at a transmembrane operating pressure of 0.20 MPa. The membrane separation process was conducted in the Technology Hall of the University of Warmia and Mazury in Olsztyn in accordance with the methodology described by Tarapata et al. (2022) [12]. The protein preparation (retentate after whey microfiltration), designated as Protein Retentate, was cooled to ±6–8 °C in an ice bath after being removed from the system and prepared for use in the experiment. The retentate had the following composition: fat content—2.01%, protein—7.55%, dry matter—14.57%, non-fat dry matter—12.67%, lactose—4.09%. The buttermilk was standardized to a protein level of 5% by adding the appropriate amount of retentate.

The Flora Danica^®^ mesophilic lactic acid bacteria culture (Novonesis, Czosnów, Poland) was used to ferment standardized buttermilk. The study also utilized the probiotic strain *Lactobacillus acidophilus* LA-5^®^ (Novonesis, Czosnów, Poland).

The reference pathogen—*Listeria monocytogenes*—was obtained from the strain collection of the Department of Food Microbiology, Meat Technology, and Chemistry at the University of Warmia and Mazury in Olsztyn. The strain was isolated from raw milk in accordance with the standards EN ISO 11290-1:2017. Identification at the species level was confirmed by MALDI-TOF MS analysis (Vitek MS, bioMérieux, Lyon, France) and subsequently confirmed by performing a standard PCR reaction [13].

### 2.2. Experiment Design

Buttermilk standardized to 5% protein using microfiltration retentate four experimental variants were prepared. The buttermilk was repasterized at 83 °C and then cooled to 30 °C. In all samples, the mesophilic starter culture Flora Danica^®^ (Novonesis, Czosnów, Poland) was used, added in accordance with the manufacturer’s instructions.

The BC (Buttermilk control) control variant consisted of buttermilk enriched with retentate and fermented using a mesophilic starter culture. In the BC-LA5 and BE-LA5-LM variants, the probiotic strain *Lactobacillus acidophilus* LA-5^®^ was additionally added to achieve an initial count of approximately 9 log CFU/mL in the product.

In two variants of buttermilk—BE-LM and BE-LA5-LM—*Listeria monocytogenes* was deliberately inoculated by adding an inoculum with a concentration of approximately 9 log CFU/mL along with the starter culture. The inoculum amount was selected to achieve an initial pathogen concentration in the product of 6 log CFU/mL. The time of pathogen introduction was defined as time 0 in the experiment. The relatively high initial inoculum level (6 log CFU/mL) was intentionally applied in accordance with challenge test approaches commonly used in food microbiology to enable reliable monitoring of pathogen survival dynamics and to assess the inhibitory potential of the tested matrix under controlled worst-case contamination conditions.

The prepared products were transferred to sterile 100-mL containers, hermetically sealed, and incubated at 25 °C until a pH of 4.6 was reached, which occurred on average after approximately 12 h of incubation. After fermentation was complete, the samples were cooled to 5, 10, and 15 °C, respectively, and stored under these conditions for the duration of the experiment, which allowed for the assessment of the dynamics of microbiological changes under conditions typical of refrigerated storage, following a break in the cold chain, and during refrigerated storage.

Microbiological and physicochemical analyses were performed at four time points: at the start of storage (0 h), and after 24, 96, 168, and 504 h of storage. The experiment was repeated three times.

### 2.3. Acidification Monitoring

The acidification kinetics of buttermilk during fermentation were monitored by measuring pH at one-minute intervals using a microelectrode connected to the Cerko Lab System (Cerko Lab, Gdynia, Poland). The measurement was conducted at 25 °C, and recording continued until a pH of 4.6 was reached, marking the end of fermentation.

### 2.4. Chemical Composition

The basic chemical composition was determined, including fat, protein, lactose, dry matter, and non-fat dry matter content, using Fourier-transform infrared (FTIR) spectroscopy with a MilkoScan™ FT1200 instrument (Foss, Hillerød, Denmark).

### 2.5. Microbiological Evaluation

All microbiological assays were performed in triplicate. Microbiological assays to determine the cell counts of lactic acid bacteria, the probiotic strain *Lactobacillus acidophilus* LA-5^®^, and the pathogen *Listeria monocytogenes* were conducted in accordance with the standards EN ISO 7218:2024, EN ISO 11290-1:2017, and EN ISO 11290-2:2017 using conventional plate counting methods.

For this purpose, the samples were homogenized and 1:10 dilutions were prepared in Peptone Salt Solution (Maximum Recovery Diluent, GranuCult^®,^ Merck, Darmstadt, Germany; cat. no. 1.12535.0500). The viable count of mesophilic lactic acid bacteria was determined by plating on M17 agar according to Terzaghi (Merck, Darmstadt, Germany; cat. no. 115108), following aerobic incubation at 30 °C for 48 h. Results were expressed as log CFU/mL. The population of the probiotic strain *Lactobacillus acidophilus* LA-5^®^ was determined on MRS medium according to de Man, Rogos, and Sharp (Merck, cat. no. 110660.0500) after incubation under anaerobic conditions at 35 °C for 72 h, using the AnaeroGen system (Oxoid, Poznań, Poland). In the pathogen-inoculated variants, the *L. monocytogenes* count was determined on selective ALOA medium (Agar *Listeria* Ottaviani & Agosti; Merck, cat. no. 1.00427.0500) in accordance with the manufacturer’s instructions. Serial decimal dilutions were prepared in Maximum Recovery Diluent, and 0.1 mL was surface-plated on ALOA agar. The limit of detection (LOD) of the method was 10 log CFU/mL. Plates were incubated at 37 °C for 24–48 h, and results were expressed as log CFU/mL.

### 2.6. Modeling Bacterial Cell Growth

The estimation of microbial cell growth within a given environmental condition of the studied product was based on kinetic parameters such as pH, temperature, and water activity (a_w_). These parameters are essential for predicting the responses of various pathogens and spoilage microorganisms, and they significantly influence critical environmental factors that affect microbial behavior. The growth modeling of the pathogenic strain *Listeria monocytogenes* was performed using the ComBase browser https://combase.errc.ars.usda.gov/ (accessed on 12 February 2026), a comprehensive database that allows for the analysis of thousands of growth and survival curves of microorganisms. These curves have been compiled from research institutions and scientific publications, providing valuable insights into the microbial dynamics under diverse environmental conditions.

The Baranyi and Roberts model was used to describe the growth curve of *Listeria monocytogenes* in this study. The model is represented by the following equation [14]:

$$\frac{dx}{dt}=\frac{q(t)}{q\left(t\right)+1}\mu \;max\left(1-{\left(\frac{x\left(t\right)}{x\;max}\right)}^{m}\right)x(t)$$

where:

- *x*(*t*) is the number of cells at time *t*,
- *x_max* is the maximum cell density,
- *m* is the parameter that characterizes the transition of the growth curve to the stationary phase,
- *q*(*t*) is the concentration of the necessary substrate, which changes over time.

This model enables the prediction of microbial growth under varying environmental conditions by incorporating substrate availability and growth dynamics [15,16].

### 2.7. Statistical Analysis

The results were statistically analyzed using STATISTICA (StatSoft Polska version 13.3) software, with a significance level of α = 0.05. Significant differences in bacterial counts under different experimental conditions were verified using one-way analysis of variance (ANOVA) and the non-parametric Bonferroni post hoc test. The non-linear least-squares minimization was performed to minimise the difference between the experimental and predicted data. The fitting accuracy was evaluated using the regression coefficient (R) and the standard deviation (SD).

## 3. Results and Discussion

**Figure 1:**
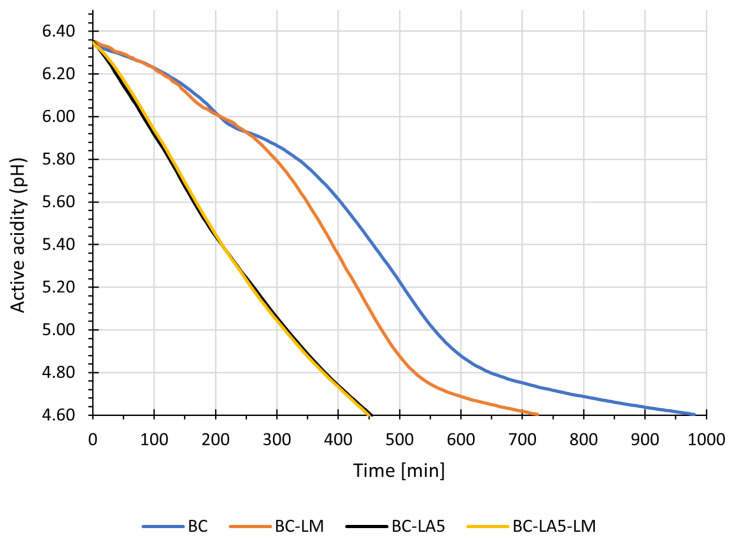
Dynamics of acidification (pH) of sweet buttermilk during production. BC—buttermilk + retentate after microfiltration of buttermilk—buttermilk control. BE-LM—buttermilk + retentate after microfiltration of buttermilk + *Listeria monocytogenes* − buttermilk experimental. BC-LA5—buttermilk + retentate after microfiltration of buttermilk + *Lactobacillus acidophilus* LA-5^®^ − buttermilk control with *Lactobacillus acidophilus* LA-5^®^. BE-LA5-LM—buttermilk+ retentate after microfiltration of buttermilk + *Listeria monocytogenes* + *Lactobacillus acidophilus* LA-5^®^ − buttermilk experimental with *Lactobacillus acidophilus* LA-5^®^.

The acidification process of milk during buttermilk production in the experiment is illustrated by the curves (Figure 1). A comparison of the acidification curves in buttermilk with the addition of *Listeria monocytogenes* (BC) showed that the presence of the pathogen in the raw material did not negatively affect the process. In buttermilk produced with only starter culture (BC), the acidification rate remained stable up to 65 min, then increased, reaching a maximum at 50–65 min, after which it decreased. For buttermilk additionally inoculated with *Listeria monocytogenes* (BE-LM), the acidification rate remained stable up to 45 min, then increased, reaching a maximum at 65 min, with pH 4.6 reached after 190 min in both cases. In probiotic buttermilk with *Lactobacillus acidophilus* added (BC-LA5, BE-LA5-LM), regardless of the presence of *L. monocytogenes*, the acidification rate remained stable up to 65 min, then increased, reaching a maximum at 75–85 min, with pH 4.6 reached after 565 min.

### Microbiological Analyses

The number of lactic acid bacteria in acidified buttermilk stored at 5, 10, and 15 °C depended on the temperature and storage time. Lactic acid bacteria in acidified buttermilk stored for 24 h reached the highest counts, amounting to 7.99 log CFU/mL and 7.97 log CFU/mL, respectively, for acidified buttermilk BC and BC-LA5.

**Figure 2:**
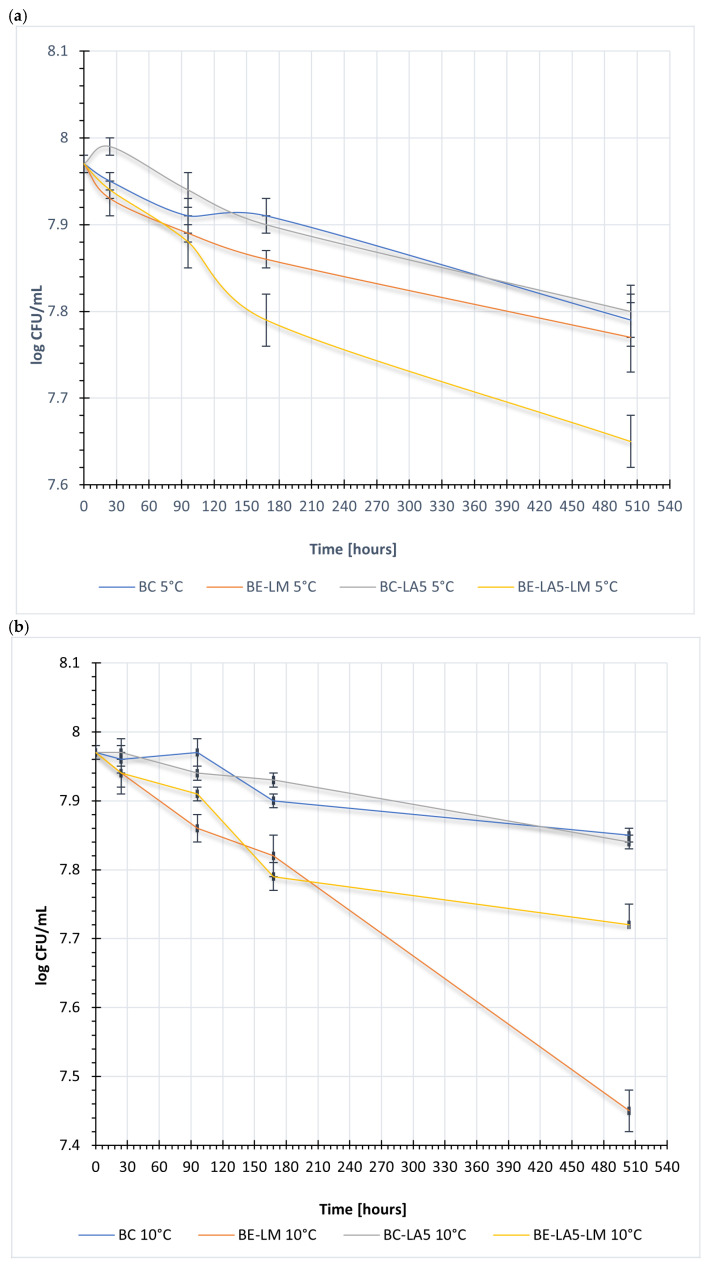

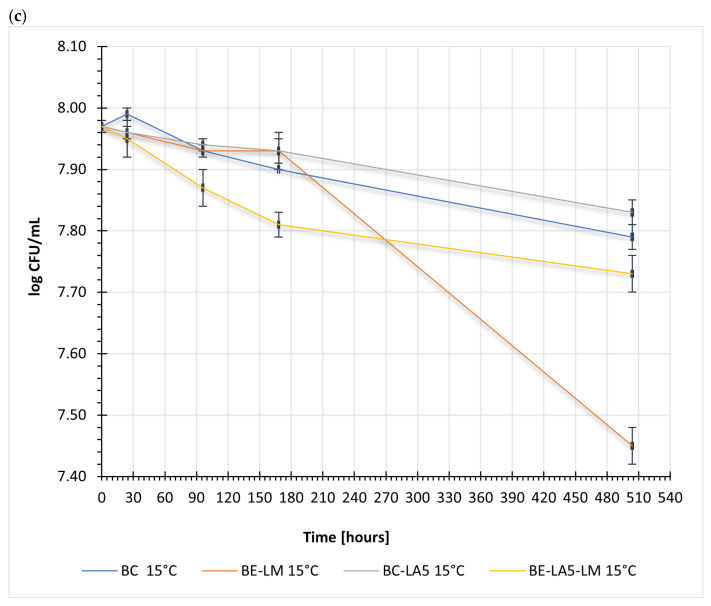
Changes in the viable counts of mesophilic streptococci in fermented buttermilk during storage at: (**a**) 5 °C, (**b**) 10 °C, and (**c**) 15 °C. BC—buttermilk + retentate after microfiltration of buttermilk—buttermilk control. BE-LM—buttermilk + retentate after microfiltration of buttermilk + *Listeria monocytogenes* − buttermilk experimental. BC-LA5—buttermilk + retentate after microfiltration of buttermilk + *Lactobacillus acidophilus* LA-5^®^ − buttermilk control with *Lactobacillus acidophilus* LA-5^®^. BE-LA5-LM—buttermilk + retentate after microfiltration of buttermilk + *Listeria monocytogenes* + *Lactobacillus acidophilus* LA-5^®^ − buttermilk experimental with *Lactobacillus acidophilus* LA-5^®^.

In samples of acidified sweet buttermilk stored at 5 °C (Figure 2a), a slower increase in the number of lactic acid bacteria was observed compared to samples stored at higher temperatures, i.e., 10 °C (Figure 2b) and 15 °C (Figure 2c).

The number of streptococcal cells gradually decreased in all tested samples starting from 96 h of storage, with the most rapid decline observed in acidified buttermilk stored at 15 °C (Figure 2c).

In all tested variants of fermented buttermilk, the number of lactic acid bacteria (LAB) counted on MRS medium remained high throughout the entire storage period. The initial LAB count was approximately 7.9–8.0 log CFU/mL, followed by a gradual decline over time, depending on the storage temperature. The highest stability of the LAB population was observed at 5 °C, while the greatest reduction in population size was observed in samples stored at 15 °C. Despite this, even after 504 h of storage, the LAB count did not fall below 7.4 log CFU/mL, confirming the good survival of lactic acid bacteria in the buttermilk matrix.

The achieved LAB level of ≥7 log CFU/mL was consistent with values obtained for fermented dairy products containing probiotic cultures and was comparable to the values reported by Ferreira et al. [17]. A significant extension of previous studies, however, was the demonstration that under the conditions of this experiment, high LAB levels persisted for at least 90 h of storage, even in the presence of *Listeria monocytogenes*. This indicates good adaptation and metabolic stability of *Lactobacillus acidophilus* LA-5^®^ in acidified buttermilk, both in samples with the addition of the pathogen and in control samples, which has not yet been unequivocally documented in the literature.

Another aspect examined was the effect of the probiotic bacterial strain *Lactobacillus acidophilus* LA-5^®^, added to the test product (fermented buttermilk), on the survival of *Listeria monocytogenes*.

**Figure 3:**
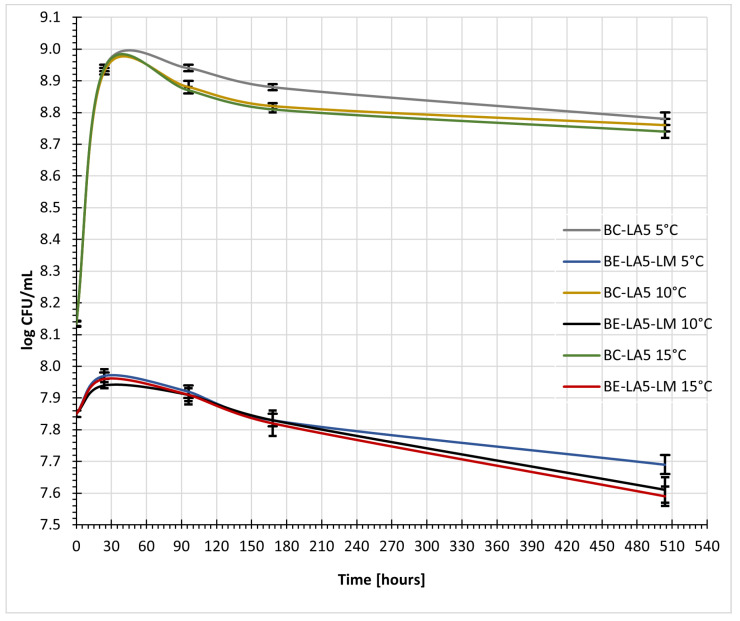
Growth in cell count of *Lactobacillus acidophilus* LA-5^®^ in fermented buttermilk in 5 °C, 10 °C, 15 °C. BC—buttermilk + retentate after microfiltration of buttermilk − buttermilk control. BE-LM—buttermilk + retentate after microfiltration of buttermilk + *Listeria monocytogenes* − buttermilk experimental. BC-LA5—buttermilk + retentate after microfiltration of buttermilk + *Lactobacillus acidophilus* LA-5^®^ − buttermilk control with *Lactobacillus acidophilus* LA-5^®^. BE-LA5-LM—buttermilk + retentate after microfiltration of buttermilk + *Listeria monocytogenes* + *Lactobacillus acidophilus* LA-5^®^ − buttermilk experimental with *Lactobacillus acidophilus* LA-5^®^.

The highest number of *Lactobacillus acidophilus* LA-5^®^ cells was observed in the samples after 24 h of storage, regardless of temperature; thereafter, a gradual decline in their number was observed during storage, which was least pronounced in the sample containing the probiotic strain. In contrast, the sample contaminated with *Listeria monocytogenes* (6 log CFU/mL) showed the fastest rate of decline in the number of *Lactobacillus* cells (Figure 3).

In all tested samples of fermented buttermilk, the population of *Lactobacillus* spp. remained stable during storage, reaching 8.7–8.8 log CFU/mL regardless of temperature, confirming the good survival and metabolic activity of lactic acid bacteria in this matrix. Previous studies indicate that buttermilk provides a favorable environment for LAB, including strains with protective potential [18]. The high survival rate of LAB may be associated with the presence of milk phospholipids, naturally occurring in buttermilk, which have been shown to enhance bacterial growth, stress resistance, and metabolic activity in dairy-based matrices [19]. Importantly, the enrichment of buttermilk with microfiltration retentate increases the availability of nutrients and bioactive components (e.g., phospholipids and MFGM fragments), which could theoretically promote the survival of *Listeria monocytogenes*. However, despite these potentially growth-supporting conditions, no increase in pathogen counts was observed. This indicates that inhibitory mechanisms associated with LAB activity—such as acidification, competition for nutrients, and production of antimicrobial metabolites—prevail over the potential growth-promoting effects of the enriched matrix.

Zhang et al. [20] demonstrated that dairy matrix composition, particularly milk fat content, significantly affected the survival of *Listeria monocytogenes* under simulated gastric conditions, highlighting the importance of matrix-specific effects beyond physicochemical parameters alone.

Statistical analysis revealed a significant effect of both the type of sample (BE-LM vs. BE-LM-LA-5), storage temperature, and incubation time on the population size of *Listeria monocytogenes* in fermented buttermilk. An analysis of variance (ANOVA) revealed significant main effects of the studied factors (*p* < 0.05) and significant interactions between storage time and the presence of *L. acidophilus* LA-5^®^, indicating that the dynamics of pathogen reduction depended simultaneously on temperature conditions and the addition of the probiotic.

In the fermented buttermilk used in this study and stored at 5 °C, no increase in the number of *Listeria monocytogenes* cells was observed in any of the tested variants. In samples inoculated with the pathogen without the presence of probiotic bacilli, a gradual reduction in population size to below the limit of detection (1 log CFU/mL) was observed after 168 h of storage. In contrast, the use of the probiotic culture *Lactobacillus acidophilus* LA-5^®^ resulted in a faster reduction in the population of *L. monocytogenes*, reaching a level below the detection limit as early as 96 h.

**Figure 4:**
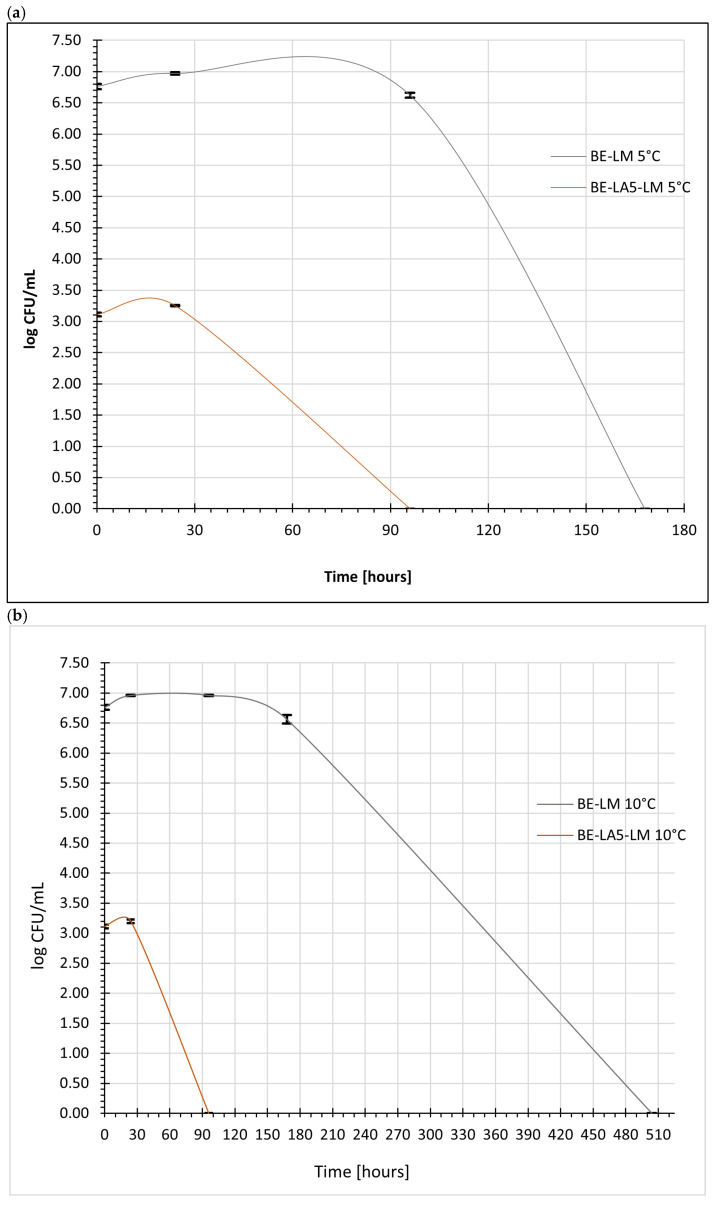

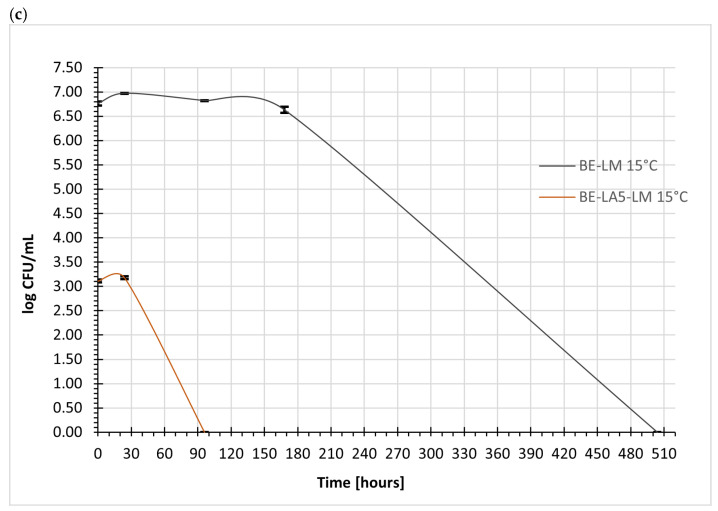
Changes in the viable counts of *Listeria monocytogenes* in fermented buttermilk during storage at (**a**) 5 °C, (**b**) 10 °C and (**c**) 15 °C. BE-LM—buttermilk + retentate after microfiltration of buttermilk + *Listeria monocytogenes* − buttermilk experimental. BE-LA5-LM—buttermilk + retentate after microfiltration of buttermilk + *Listeria monocytogenes* + *Lactobacillus acidophilus* LA-5^®^ − buttermilk experimental with *Lactobacillus acidophilus* LA-5^®^.

These results confirm the effectiveness of fermented buttermilk as a medium that limits the survival of *L. monocytogenes* under refrigerated conditions and highlight the significant role of bioprevention involving the probiotic lactic acid bacteria *L. acidophilus* LA-5^®^ (Figure 4a). The observed inhibition of *Listeria monocytogenes* may be attributed to the combined effect of low pH, organic acid production, and microbial competition exerted by LAB. Additionally, the production of antimicrobial metabolites such as bacteriocins and hydrogen peroxide may further contribute to the inhibitory effect.

No increase in the number of *Listeria monocytogenes* cells was observed in fermented buttermilk stored at both 10 °C and 15 °C. In samples inoculated with the pathogen, its count reached 3 and 7 log CFU/mL in the BE-LA5-LM (with probiotic bacilli) and BE-LM (without probiotic bacilli) samples, respectively, during the initial storage period. In both variants, a reduction in the number of *L. monocytogenes* was observed, the rate of which was faster in the presence of *L. acidophilus* LA-5^®^ (after 96 h) than in the BE-LM variant without probiotic rods (after 504 h).

These differences were statistically significant in most of the analyzed time-temperature combinations (*p* < 0.05), and the effect sizes indicated a moderate to large impact of the probiotic culture addition on reducing pathogen survival. The results suggest that the observed differences are not due to random measurement variability but are the result of the experimental factor under study.

These results confirmed the effectiveness of fermented buttermilk as a medium that inhibits the survival of *L. monocytogenes* and indicate the significant role of the probiotic *L. acidophilus* LA-5^®^ in reducing the population of *L. monocytogenes* in fermented buttermilk under conditions of elevated storage temperature (Figure 4b,c).

There was a significant interaction between the storage temperature and the presence of *L. acidophilus* LA-5^®^, indicating that the bioprotective effect increased as the storage temperature rose. From a microbiological safety perspective, this means that fermented buttermilk does not provide an environment conducive to the growth of *L. monocytogenes*, and the presence of the probiotic strain further enhances this inhibitory effect.

The observed reduction in the population of *L. monocytogenes* in fermented buttermilk can be interpreted as the result of environmental barriers and bioprevention, in line with the approach used in shelf-life studies and challenge tests for ready-to-eat foods [21].

The counts of lactic acid bacteria and lactic streptococci in our study were comparable to the results obtained in a pilot study by Silva et al. for buttermilk produced from sheep’s milk [22].

Studies conducted by Pereira et al. [23] showed that in buttermilk samples containing probiotics and bio-protective cultures, the highest numbers of lactic acid bacteria and lactococci were at a level of approximately 7–9 log CFU/mL throughout the entire storage period. The observed counts were above the values indicated as necessary to confirm the probiotic properties of a food product (>7 log CFU/mL) for *Lactobacillus*.

In all variants containing the probiotic strain, the number of *L. acidophilus* LA-5^®^ bacteria exceeded 7 log CFU/mL during the 504-h storage period. Our own research has shown that probiotics can exhibit bioprotective properties, which is why they are desirable in the production of acidified buttermilk, as they maintain high levels throughout the entire storage period. Furthermore, the results obtained in the present study are consistent with the observations of Fernandes et al. [24], who demonstrated that the coexistence of *L. innocua* and *L. acidophilus* LA-5^®^ in dairy products limits the growth of both microorganisms during storage [25,26].

## 4. Predictive Growth (COMBASE)

Predictive microbiology is increasingly used as a supportive tool in food safety assessment, including in the context of recent regulatory updates concerning *Listeria monocytogenes* in ready-to-eat products [27,28]. Such models enable estimation of microbial behavior under defined physicochemical conditions and are commonly applied in risk assessment and process optimization.

**Figure 5:**
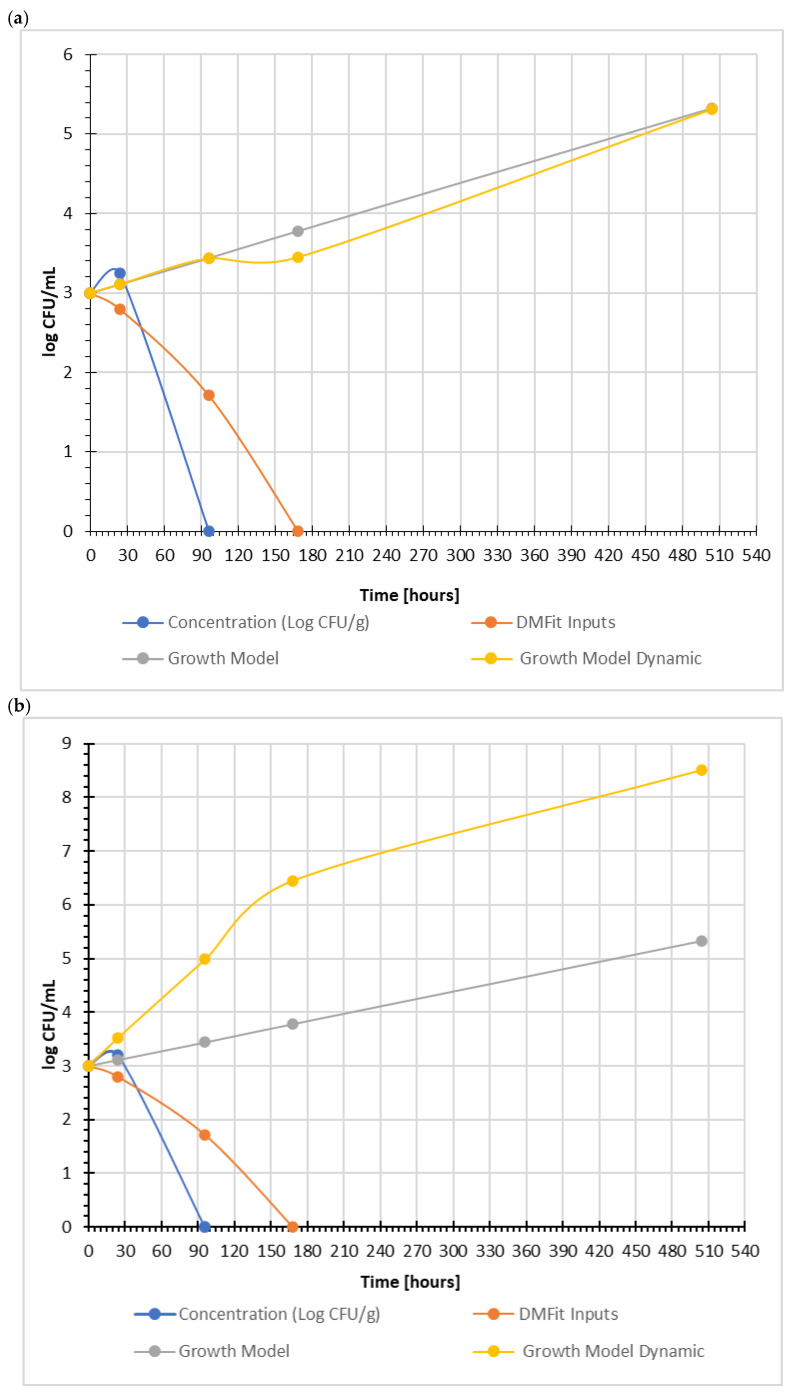

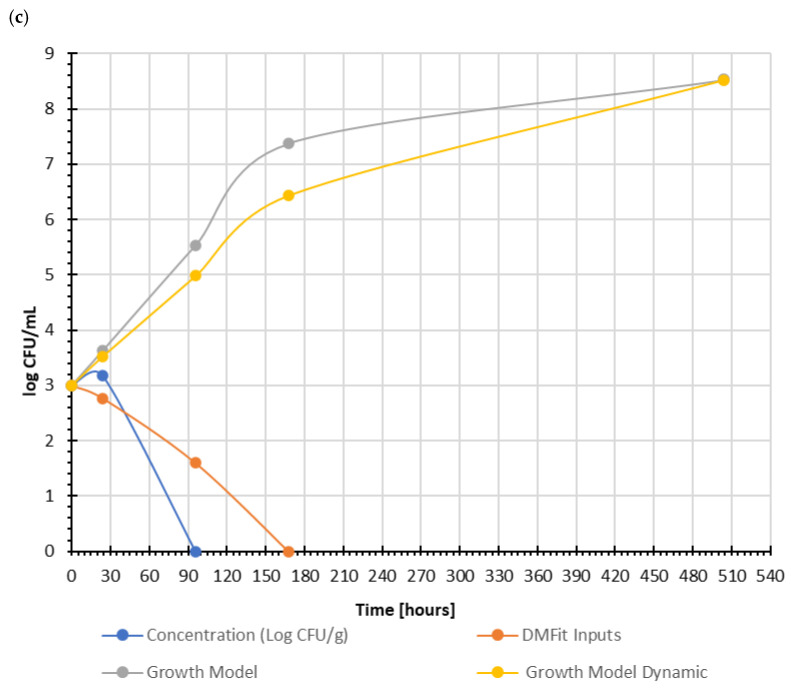
Prediction of the growth of *Listeria monocytogenes* cells in acidified buttermilk at (**a**) 5 °C, (**b**) 10 °C, and (**c**) 15 °C. Concentration (log CFU/mL)—actual bacterial concentration values for YE-LA5-LM (buttermilk + retentate after microfiltration of buttermilk + *L. monocytogenes* + *L. acidophilus* LA-5^®^) DMFit Inputs—data obtained from the experiment. Growth Model—predicted growth model for the pathogen *Listeria monocytogenes* (*takes constant environmental conditions into account*). Growth Model Dynamic—dynamic growth model of the pathogen *Listeria monocytogenes* (*takes variable environmental conditions into account*).

However, a clear discrepancy between model predictions and experimental data was observed across all temperatures. As shown in Figure 5a (5 °C), both predictive models indicated a gradual increase in pathogen counts over time, whereas the experimental data (DMFit Inputs and measured concentration) showed a marked and continuous decline, with pathogen levels approaching the detection limit during storage.

In the present study, predictive modeling using ComBase indicated the potential for growth of *Listeria monocytogenes* under the tested conditions (5–15 °C), based on input parameters such as temperature, pH, and water activity. Both static (Growth Model) and dynamic (Growth Model Dynamic) simulations predicted an increase in pathogen population following the lag phase (Figure 5a–c).

A similar pattern was observed at 10 °C (Figure 5b), where the dynamic model predicted rapid growth and the static model a moderate increase, while the experimental data again demonstrated a strong reduction in *L. monocytogenes* counts.

At 15 °C (Figure 5c), the divergence between predicted and observed behavior was even more pronounced. The models predicted substantial growth of the pathogen, reaching high population levels, whereas the experimental data showed rapid inactivation and a complete decline of detectable cells within the early stages of storage.

This consistent mismatch between predicted and observed behavior represents a key finding of the study. The results indicate that predictive models may overestimate the growth potential of *L. monocytogenes* in complex, biologically active food matrices such as fermented buttermilk [29].

Predictive models used in food microbiology are based on mathematical descriptions of microbial population dynamics and are increasingly supported by advanced computational tools and databases. These models enable rapid estimation of microbial behavior under defined physicochemical conditions and are widely applied in risk assessment and process design.

However, predictions generated by such models should be interpreted with caution. They are typically based on simplified assumptions and data derived from laboratory media and therefore may not fully reflect the complexity of real food systems. Consequently, model outputs should always be validated against experimental data and interpreted within the context of current microbiological knowledge.

In the context of the analyses discussed here, it is important to distinguish between two types of predictive models: static and dynamic. Static models assume constant environmental conditions (e.g., temperature, pH, water activity), which simplifies calculations but may limit their applicability to real systems. In contrast, dynamic models account for changes in environmental parameters over time, providing a more realistic representation of microbial behavior in food products [30,31].

The observed discrepancy may be related to the fact that predictive microbiology models are commonly developed using simplified laboratory media and therefore may not fully account for the complexity of real food systems. In fermented buttermilk, factors such as the presence of metabolically active lactic acid bacteria, accumulation of organic acids, microbial competition, and matrix-specific components, including milk phospholipids and milk fat globule membrane (MFGM) fragments, may substantially influence pathogen behavior and survival.

Similar limitations of predictive microbiology models were highlighted by Gowda et al. [32], who reported that models developed using laboratory media frequently overestimate the growth potential of *Listeria monocytogenes* when applied to real food matrices. According to the authors, broth-based models do not adequately reflect matrix complexity, microbial interactions, nutrient mobility, or the influence of background microbiota, all of which may significantly affect pathogen behavior in food systems.

Recent studies have also emphasized that interactions between lactic acid bacteria and *Listeria monocytogenes* in food systems are highly complex and cannot be explained solely by physicochemical parameters. Tarlak et al. [33] demonstrated that co-culture interactions between LAB and *L. monocytogenes* in milk systems required advanced modelling approaches, including machine learning-assisted models, to adequately describe microbial behavior and competitive dynamics. These findings support the interpretation that conventional predictive microbiology models may not fully capture the biological interactions occurring in fermented dairy matrices.

Therefore, the observed reduction of *L. monocytogenes* cannot be explained solely by physicochemical parameters and highlights the limitations of predictive models when applied to biologically active systems. In this context, predictive modeling should be considered an exploratory tool rather than a confirmatory one, and its results require experimental validation.

It should be noted that the present study was designed as a product-level challenge study rather than a mechanistic investigation. Consequently, the relative contribution of individual inhibitory factors—including fermentation, retentate enrichment, viable LAB cells, organic acids, bacteriocins, and other antimicrobial metabolites—cannot be distinguished [34,35]. The absence of non-fermented controls, retentate-free variants, LA-5-only acidification controls, and cell-free supernatant assays represents a limitation of this study.

The obtained results are consistent with challenge test approaches applied to ready-to-eat foods, where the absence of pathogen growth is considered a key safety criterion. Overall, the findings demonstrate that the behavior of *L. monocytogenes* in complex food systems cannot be predicted solely based on physicochemical parameters, emphasizing the need for experimental validation of predictive microbiology models.

## 5. Conclusions

The results of this study demonstrated that fermented buttermilk enriched with microfiltration retentate did not support the growth of *Listeria monocytogenes* under the tested storage conditions. Instead, a gradual reduction in pathogen counts was observed at all analyzed temperatures.

The presence of the probiotic strain *Lactobacillus acidophilus* LA-5^®^ was associated with a faster reduction of *L. monocytogenes* compared to variants without the probiotic culture. At the same time, high viability of lactic acid bacteria was maintained throughout the storage period, confirming good adaptation of LAB to the buttermilk matrix.

Predictive modeling using ComBase indicated the potential for pathogen growth under comparable physicochemical conditions, whereas experimental data demonstrated a consistent reduction in *L. monocytogenes* counts. This discrepancy highlights the limitations of predictive microbiology models when applied to complex, biologically active food matrices and emphasizes the importance of experimental validation.

The observed inhibitory effect may be associated with factors such as acidification, microbial competition, and LAB metabolic activity; however, these mechanisms were not directly investigated in this study.

Overall, the obtained results indicate that fermented buttermilk enriched with retentate and supplemented with probiotic LAB may provide conditions limiting the survival of *L. monocytogenes*. The study also contributes to a better understanding of pathogen behavior in fermented dairy systems and the applicability of predictive microbiology tools in such matrices.

## Data Availability

The original contributions presented in this study are included in the article. Further inquiries can be directed to the corresponding author.
